# Dialysis with high-flux membranes significantly affects plasma levels of neutrophil gelatinase-associated lipocalin

**DOI:** 10.1186/s13054-016-1198-4

**Published:** 2016-01-31

**Authors:** Carlo Donadio

**Affiliations:** Department of Clinical and Experimental Medicine, Division of Nephrology, University of Pisa, Via Savi 10, 56100 Pisa, Italy

I read with interest the recent article by Schilder et al. reporting that plasma levels and the biomarker value of neutrophil gelatinase-associated lipocalin (NGAL) in critically ill patients with acute kidney injury (AKI) are not affected by continuous venovenous hemofiltration (CVVH) [1]. Recently, Honore et al. [2] commented on the data by Schilder et al., suggesting that further studies are warranted to definitely assess the membranes and the dialytic techniques that can remove NGAL from plasma and thus affect its accuracy as a marker of AKI.

Our results in 31 patients on maintenance hemodialysis (MHD), published in *Critical Care* as part of a study that evaluated the effect of glomerular filtration rate impairment on diagnostic performance of NGAL [3], appear quite different from those found by Schilder et al. in critically ill patients who received CVVH. Patients on MHD received low-flux dialysis (23 treatments) with a polysulfone membrane (F8; Fresenius, Bad Homburg, Germany), or different high-flux membranes. High-flux dialysis treatments were performed in 13 patients by using a triacetate cellulose membrane with a surface of 1.9 m^2^ and an ultrafiltration rate (UFR) of 8474 mL/h per 100 mm Hg (N190 FH; Nipro, Osaka, Japan). The remaining eight treatments were performed as hemodiafiltration with a polyphenylene membrane with a surface of 2.0 m^2^ and a UFR of 6800 mL/h per 100 mm Hg (Phylther; Bellco, Mirandola, Italy) or as an acrylonitril and natrium metallylsulfone copolymer membrane with a surface of 2.15 m^2^ and a UFR of 6500 mL/h per 100 mm Hg (Nephral 500; Gambro, Lund, Sweden). Dialysis length was 4.0 ± 0.2 h, and blood flow was 312 ± 43 mL/min. Mean plasma concentrations of NGAL increased by 9.1 ± 24.4 % at the end of low-flux dialysis, indicating that low-flux polysulfone did not remove NGAL (Fig. 1). Treatments with high-flux membranes decreased plasma NGAL significantly (*P* < 0.0001). The reduction ratio of NGAL was higher after hemodiafiltration (52.1 ± 26.7 %) than after high-flux dialysis (26.6 ± 26.1 %, *P* = 0.053).

**Fig. 1:**
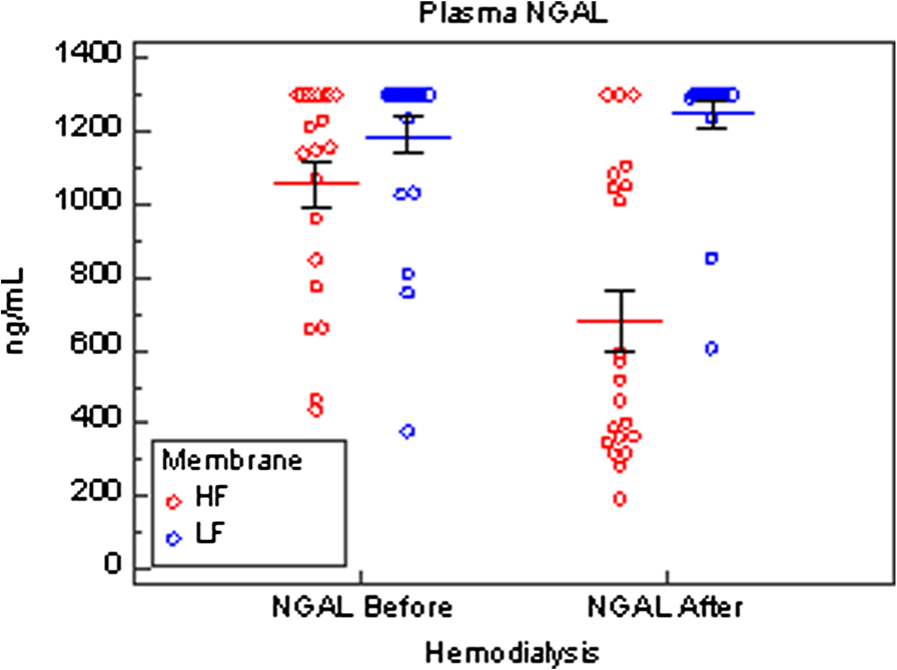
Effect of dialysis with low-flux (LF) or high-flux (HF) membranes on plasma concentrations of neutrophil gelatinase-associated lipocalin (NGAL) in 31 patients on maintenance hemodialysis

Schilder et al. performed CVVH treatments with a triacetate membrane with a surface of 1.9 m^2^ (UF-205), a UFR of 3700 mL/h per 100 mm Hg lower than that of our triacetate membrane, and a lower sieving coefficient for middle molecules (0.81 versus 0.91 for myoglobin). During treatments, the blood flow was kept at 180 mL/min and the substitution fluid at 2 L/h. These differences can justify, at least in part, the lower removal of NGAL found in patients with AKI than in patients on MHD.

Besides the chemical composition of the membrane, different parameters of the dialysis session may influence the removal of NGAL. Therefore, further studies are warranted to assess the membranes and the dialytic techniques that can affect the accuracy of NGAL as a marker of AKI.

## Abbreviations

AKI: Acute kidney injury
CVVH: Continuous venovenous hemofiltration
MHD: Maintenance hemodialysis
NGAL: Neutrophil gelatinase-associated lipocalin
UFR: Ultrafiltration rate
